# Myricetin Exerts Antibiofilm Effects on *Candida albicans* by Targeting the RAS1/cAMP/EFG1 Pathway and Disruption of the Hyphal Network

**DOI:** 10.3390/jof11050398

**Published:** 2025-05-21

**Authors:** Melda Meral Ocal, Merve Aydin, Esra Sumlu, Emine Nedime Korucu, Ali Ozturk

**Affiliations:** 1 Department of Biotechnology, Faculty of Science, Mersin University, Mersin 33343, Turkey; meldameralocal@mersin.edu.tr; 2 Department of Medical Microbiology, Faculty of Medicine, Erzincan Binali Yildirim University, Erzincan 24100, Turkey; 3 Department of Medical Microbiology, Faculty of Medicine, KTO Karatay University, Konya 42020, Turkey; 4 Department of Medical Pharmacology, Faculty of Medicine, KTO Karatay University, Konya 42020, Turkey; esra.sumlu@karatay.edu.tr; 5 Department of Molecular Biology and Genetics, Faculty of Science, Necmettin Erbakan University, Konya 42090, Turkey; enkorucu@erbakan.edu.tr; 6 Department of Medical Microbiology, Faculty of Medicine, Nigde Omer Halisdemir University, Nigde 51240, Turkey; ozturkali@ohu.edu.tr

**Keywords:** myricetin, *Candida albicans*, antifungal activity, biofilm-related genes, FESEM, *Galleria mellonella*

## Abstract

Increasing antifungal resistance and side effects of existing drugs demand alternative approaches for treating *Candida* (*C.*) infections. This study aimed to comprehensively evaluate the antifungal efficacy of myricetin (MYR), a natural flavonoid, against both fluconazole (FLC)-resistant and susceptible clinical *Candida* strains, with a particular focus on its inhibitory effects on *C. albicans* biofilms. Antifungal susceptibility was evaluated on *Candida* spp. by the broth microdilution method, and the impact of myricetin on *C. albicans* biofilms was determined using the Cell Counting Kit-8 (CCK-8) assay. To understand the molecular mechanisms underlying the antibiofilm properties of myricetin, expression analysis of genes in the RAS1/cAMP/EFG1 pathway (*ALS3*, *HWP1*, *ECE1, UME6*, *HGC1*) and cAMP-dependent protein kinase regulation (*RAS1, CYR1, EFG1*) involved in the transition from yeast to hyphae was performed. Field emission scanning electron microscopy (FESEM) was used to study the ultrastructural changes and morphological dynamics of *Candida* biofilms after exposure to MYR and FLC. The in vivo toxicity of myricetin was evaluated by survival analysis using the *Galleria mellonella* model. Myricetin significantly suppressed key genes related to hyphae development (*RAS1*, *CYR1*, *EFG1*, *UME6*, and *HGC1*) and adhesion (*ALS3* and *HWP1*) in both clinical and reference *Candida* strains at a concentration of 640 µg/mL. FESEM analysis revealed that myricetin inhibited hyphae growth and elongation in *C. albicans*. This study highlights the promising antibiofilm potential of myricetin through a significant inhibition of biofilm formation and hyphal morphogenesis.

## 1. Introduction

*Candida* (*C*.) species, commensal fungi residing in human mucosal surfaces and skin that can become virulent under immunocompromised conditions or microbiota imbalances, causing infections from superficial mucosal candidiasis to systemic candidemia [1,2,3]. The global burden of *Candida* infections has risen due to increased immunosuppressive therapies and invasive medical procedures, and *C. albicans* accounts for nearly 50% of global candidemia cases [4,5,6].

*Candida albicans* virulence is marked by its ability to form complex, drug-resistant biofilms on biotic and abiotic surfaces. These biofilms comprise yeast, pseudohyphal, and hyphal cells within a self-produced extracellular matrix (ECM) [7,8,9]. The matrix provides structural stability and acts as a barrier against antifungals and host immune responses, leading to multidrug resistance and persistence in clinical settings [8,9,10]. The yeast-to-hyphae transition, regulated by the RAS1-cAMP-EFG1 pathway, drives biofilm formation and enables tissue invasion [11,12,13].

The limitations of current antifungal therapies, particularly against biofilm-embedded cells, necessitate the urgent development of safe and effective antibiofilm agents. Traditional antifungals, such as azoles and echinocandins, often fail to eliminate biofilm infections and may contribute to the development of resistant strains [14,15,16]. Consequently, plant-derived natural compounds are gaining attention as potential alternatives due to their broad bioactivities, low toxicity, and cost-effectiveness [16,17].

Myricetin (MYR, 3,5,7,3′,4′,5′-hexahydroxyflavonol), a naturally occurring flavonoid found in plant families such as *Myricaceae*, *Vitaceae*, *Leguminosae*, and *Rosaceae*, has shown promising antifungal potential [18,19]. Although limited studies exist on its antifungal activity, it has been reported that a combination of MYR and farnesol significantly suppressed water-soluble exopolysaccharides in the ECM and inhibited biofilm formation in cariogenic dual-biofilms of *Streptococcus mutans* and *C. albicans* [20]. MYR/miconazole thermosensitive hydrogels have been shown to significantly inhibit *C. albicans* biofilm and the expression of *TPK2*, *EFG1*, and *HST7*, key genes involved in biofilm formation [21]. Myricetin’s multiple hydroxyl groups structurally enable interactions with fungal cell wall components and signaling pathways, though its precise molecular mechanisms remain not fully understood [18,22].

This study aimed to evaluate the antifungal efficacy of MYR against both FLC-resistant and susceptible clinical *Candida* strains under planktonic conditions, as well as its inhibitory effect on *C. albicans* biofilms using the CCK-8 assay, in comparison with FLC. Myricetin’s impact on the expression levels of genes involved in adhesion, yeast-to-hypha transition, and biofilm formation was assessed using qPCR. To further elucidate the morphological and ultrastructural changes in *C. albicans* biofilms, field emission scanning electron microscopy (FESEM) was employed. Furthermore, MYR in vivo toxicity and therapeutic efficacy were evaluated using a *Galleria mellonella* larvae model, providing insights into its potential as a safe and effective antifungal agent. These comprehensive analyses aimed to shed light on the therapeutic potential of MYR in combating fungal infections, particularly those involving biofilm formation and FLC-resistant strains.

## 2. Materials and Methods

### 2.1. Candida Strains and Chemicals

The 28 clinical *Candida* strains used in this study were obtained from the Culture Collection of Nigde Ömer Halisdemir University Faculty of Medicine, Medical Mycology Laboratory. *C. albicans* (ATCC 10231), *C. glabrata* (ATCC 90030), *C. krusei* (ATCC 6258), and *C. parapsilosis* (ATCC 22019) standard strains used for quality control were obtained from the American Type Culture Collection (ATCC). The strains were identified to species level according to their growth characteristics on colony morphology, performing a germ tube test, and analyzing microscopic morphology on cornmeal Tween 80 agar and VITEK-2 ID YST system (BioMérieux, Marcy l’Etoile, France). All *Candida* strains were stored in a glycerol medium at −80 °C. Before each experiment, the strains were routinely cultured on Sabouraud Dextrose Agar (SDA; Neogen Corporation, Lansing, MI, USA) at 35 °C for 24–48 h and subcultured at least twice on SDA medium to ensure purity and activity.

The chemicals used in this study were fluconazole by Target Mol (Wellesley Hills, MA, USA; 99.17%; CAS 86386-73-4) and myricetin by ChemCruz (Dallas, TX, USA; 98%; CAS 529-44-2). The MYR and FLC stock solutions were prepared by diluting dimethyl sulfoxide (DMSO). These stock solutions were serially diluted in bicarbonate and L-glutamine-free RPMI 1640 (Gibco, Billings, MT, USA) containing MOPS (AppliChem, Darmstadt, Germany). This procedure allowed two-fold serial dilutions ranging from 2560 to 5 μg/mL for MYR and 256 to 0.5 μg/mL for FLC, all in RPMI 1640 medium containing 0.1% DMSO.

### 2.2. Antifungal Susceptibility

Antifungal susceptibility tests for MYR and FLC were conducted using the broth microdilution method, following CLSI M27-A3 guidelines [23]. Myricetin (2560–0.5 μg/mL) and FLC (256 to 0.5 μg/mL) serial dilutions were added to the test wells. The strains were incubated in SDA for 24 h at 35 °C and then subcultured. Following the adjustment of the cell density to 0.5 McFarland, the yeast suspension was added to the wells at a final density of 0.5 × 2.5 × 10^3^ CFU/mL and incubated at 35 °C for 24 h. The minimum inhibitory concentration (minimum inhibitory concentration, MIC_50_) values for MYR and FLC were determined visually by two blinded observers and defined as the lowest concentration of the drugs that resulted in 50% growth inhibition as relative to the growth of the control well. The FLC resistance breakpoints were evaluated according to CLSI M27-A3 guidelines. This assay was performed in triplicate for each strain, and to exclude the possible influence of solvent activity, a control containing only DMSO was prepared.

### 2.3. Biofilm Formation and Inhibition Assay

#### 2.3.1. Biofilm Biomass Determination by Crystal Violet Staining

The ability of *C. albicans* strains to form biofilms was performed by the Crystal violet (CV) assay in 96-well microplates, as described previously [24]. Briefly, a cell suspension containing 1 × 10⁶ cells/mL was prepared from *C. albicans* isolates, and 200 µL was transferred into the wells. The plates were incubated at 37 °C for 90 min, and non-adherent cells were removed three times, washing with 1× PBS. 200 μL of MOPS-buffered RPMI 1640 medium was added to each well, incubated at 37 °C for 24 h, and washed twice with 1× PBS after removing the supernatant. The microplate was dried at 60 °C for 30 min and then stained with 1% *w*/*v* crystal violet solution (Carlo Erba Reagents, Milan, Italy) for 15 min. After removing excess dye by washing with 1× PBS, the remaining dye in the biofilms was dissolved by adding 150 μL of 96% (*v*/*v*) ethanol. The biofilm biomass was then determined using a Multiskan Sky ELISA reader (Thermo Scientific™, Waltham, MA, USA) based on absorbance measurements at 595 nm. Results were expressed as the average of at least three replicates. The strains were classified into the following four categories: non-adherent weak, moderate, and strong biofilm producers, according to Stepanović et al. [25].

#### 2.3.2. Investigation of the Effect of MYR on *Candida* Strain Biofilms by CCK-8 Test

The cytotoxic activity of MYR and FLC on *C. albicans* biofilm was measured using SuperKine™ Maximum Sensitivity Cell Counting Kit-8 (CCK-8, Abbkine, GA, USA). *Candida albicans* biofilms were established in 96-well microplates as described above. To determine the effect of myricetin on *C. albicans* biofilms, the strong biofilm-forming *C. albicans* strains were used. Briefly, *C. albicans* suspension was adjusted in RPMI-1640 medium (1 × 10⁶ cells/mL) and transferred into wells. After 90 min for cell adhesion, the wells were washed with PBS to remove non-adherents. After washing, to ensure that adhesion to the microplate well surface was achieved, the microscopic examination of the biofilms formed on the microplates was performed using a Nikon TS2 inverted microscope (Nikon Instruments Inc., Tokyo, Japan). The plates were incubated at 37 °C for 24 h with different concentrations of myricetin and FLC, ranging from 2560–0.5 μg/mL and 256–0.5 μg/mL. The supernatant was then removed, and 100 µL RPMI 1640-MOPS medium and 10 µL CCK-8 solution (5 mg/mL) were added to each well and incubated at 37 °C for 4 h. The optical density of each well was measured at 450 nm using a Multiskan Sky (Thermo Fisher Scientific, Waltham, MA, USA) ELISA reader. The absorbance value of the blank well was subtracted from the absorbance values of the test wells. Three replicates of each assay were performed to ensure the accuracy and consistency of the results.

### 2.4. RNA Isolation, cDNA Synthesis, and qRT-PCR Expression Analysis

In this study, to investigate the effects of MYR on gene activation/repression associated with the biofilm formation of *C. albicans*, *C. albicans* ATCC 10231 biofilms were prepared according to Section 2.3. with and without 640 µg/mL MYR and 16 µg/mL FLC. The well untreated with MYR and FLC was considered the positive control, while the well containing only culture medium was regarded as the negative control. After incubation at 37 °C for 24 h, the biofilm was washed with 1× PBS. After cell viability was confirmed using CCK8, RNA was extracted from *C. albicans* cells using a Yeast RNA Kit (Zymo Research, Irvine, CA, USA), following the manufacturer’s recommendations. The concentration and purity of the extracted RNA were evaluated using a Multiskan Sky μDrop™ plate (Thermo Fisher Scientific, Waltham, MA, USA). Complementary DNA (cDNA) was then synthesized using a commercial cDNA synthesis kit (IScript cDNA synthesis kit, Bio-Rad, Hercules, CA, USA). Real-time PCR was performed using the FastStart Essential DNA Green Master Mix kit (Roche, Basel, Switzerland) according to the manufacturer’s protocol. For each gene, the PCR mix was prepared in a final volume of 20 μL containing 10 μL 2× SYBR Green Master Mix (Roche, Basel, Switzerland), 5 μL cDNA template, 2 μL primer mix (at 0.5 mM concentration), and 3 μL nuclease-free water. Primers targeting genes in the RAS1/cAMP/EFG1 pathway (*ALS3*, *HWP1*, *ECE1*, *UME6*, *HGC1*), cAMP-dependent protein kinase regulation (*RAS1*, *CYR1*, *EFG1*) for yeast to hyphae transition are given in Table 1. *ACT1* was used as the housekeeping gene for normalization of gene expression. Quantitative real-time PCR (qRT-PCR) was performed and analyzed using QuantStudio™ 3 (Thermo Fisher Scientific, Waltham, MA, USA) with the following cycle conditions: 10 min at 95 °C for initial denaturation, followed by 40 cycles of denaturation at 95 °C for 10 s and extension at 72 °C for 15 s. Each experiment was performed in triplicate and conducted as three independent experiments. At the end of each reaction, amplification plots and melting temperature curves were reviewed, and relative fold changes in gene expression levels were analyzed using the 2^−ΔΔCt^ method previously described.

### 2.5. FESEM Analysis

To investigate the effect of MYR and FLC on *Candida* biofilm architecture and morphological characteristics, *C. albicans* (ATCC 10231) cell suspension prepared as 1 × 10^6^ cells/mL in RPMI-1640 medium was added to 6-well microplates containing special polystyrene discs (1 cm^2^). The wells were incubated at 37 °C for 120 min to allow cell adhesion. The wells were washed with sterile 1× PBS to remove non-adherent cells. Yeast suspension and 640 µg/mL MYR and 16 µg/mL FLC concentration were added and incubated at 37 °C for 24 h. After the incubation period, 6-well microplates containing the discs were washed three times with PBS. Glutaraldehyde (2% vol/vol), Tekkim, Bursa, Turkey) stabilized overnight, followed by a dehydration step. Dehydration was performed using an ethanol series of 10%, 25%, 50%, 75%, and 90% ethanol for 10 min each and 100% for 30 min. The coverslips were dried overnight and coated with iridium with a sputter coater (Leica EM ACE600, Wetzlar, Germany). Biofilms were visualized by an emission scanning electron microscope (Zeiss Gemini500, Oberkochen, Germany) at magnifications ranging from 1000× to 10,000×.

### 2.6. In Vivo Relative Toxicity Evaluation with Galleria mellonella Larvae Model

MYR’s in vivo toxicity and efficacy were analyzed using the invertebrate animal model *Galleria* (*G.*) *mellonella*. The larvae of *G. mellonella* were grown in the Medical Microbiology Laboratory of Nigde Ömer Halisdemir University Faculty of Medicine in a synthetic medium at 30 °C and 65% humidity under a 12-h light/dark cycle. The larvae in the last larval stage, approximately 2–3 cm long and weighing 200–400 mg, were used in the experiments. Briefly, overnight cultures of *C. albicans* (ATCC 10231) were grown in 5 mL YPD medium for 18 h at 35 °C, washed twice, and resuspended in PBS. Cell suspensions were prepared in saline at a final 1 × 10⁶ cells/mL concentration.

The larvae were randomly divided into six groups (10 larvae/group), as follows: (1) Untreated healthy control group, (2) PBS-control group, (3) Needle-prick control group, (4) Untreated infection group, (5) Myricetin-treated group, (6) FLC-treated group.

A yeast suspension (10 µL) was injected into the left prolegs of larvae. In contrast, a Hamilton syringe injected myricetin (640 μg/mL) and FLC (16 μg/mL) into the right prolegs two hours after infection. Following injection, the larvae were incubated at 37 °C and monitored every 24 h for 10 days. All larval groups were incubated at 35 °C for 10 days. For survival curve analysis, the survival rate was recorded every 24 h and analyzed by the Kaplan–Meier method. At the end of the experiment, the hemolymph of infected larvae was microscopically examined and cultured on SDA to confirm the presence of yeasts.

### 2.7. Statistical Analysis

Statistical analysis was conducted using GraphPad Prism 9 (GraphPad Software Inc., San Diego, CA, USA). To evaluate differences between groups, we employed an analysis of variance (ANOVA) followed by a Bonferroni post hoc test. Significant results are presented as the mean plus the standard deviation (SD). Survival curves for *Galleria mellonella* were generated using the Kaplan–Meier method and analyzed through the log-rank (Mantel–Cox) test. A *p*-value of less than 0.05 was considered statistically significant.

## 3. Results

### 3.1. Antifungal Activity of Myricetin Against Candida Species

Myricetin showed antifungal activity at 320 μg/mL in clinical strains of *C. kefyr*, *C. krusei*, *C. lusitaniae*, and *C. spherica*. Low MIC_50_ values for MYR (40 μg/mL) were detected in *C. parapsilosis* ATCC 22019 and *C. glabrata* ATCC 90030, as well as clinical strains of *C. glabrata.* For *C. albicans* ATCC 10231 and *C. krusei* ATCC 6258 strains, MIC_50_ values of MYR 320 μg/mL and 80 μg/mL were detected, respectively. In *C. albicans* and *C. tropicalis* strains, MIC_50_ values varied in a wide range between 40 and 640 μg/mL, regardless of FLC susceptibility (Table S1). Antifungal susceptibility test results of *Candida* spp. against MYR and FLC are given in Table 2.

### 3.2. Biofilm-Forming Ability of Candida albicans Strains

**Figure 1:**
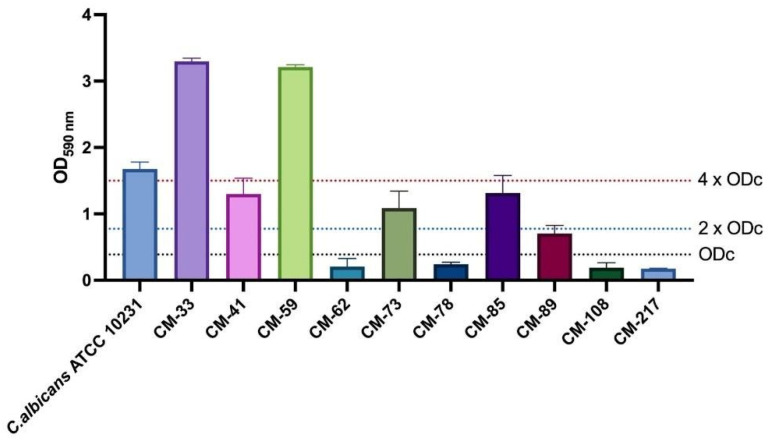
OD values of *C. albicans* strains.

OD values of all *C. albicans* strains used in this study are shown in Figure 1, and the OD cut-off value (ODc) was calculated as 0.38. According to the classification of Stepanovic et al. [25], it was observed that CM-62, CM-78, CM-108, and CM-217 strains were not adherent, CM-89 produced weak biofilm, and CM-41, CM-73, and CM-85 produced moderate biofilm. As *C. albicans* ATCC 10231, CM-33, and CM59 strains produced strong biofilms, these strains demonstrated the antibiofilm activity by CCK-8 analysis and expression analysis of biofilm-related genes by qPCR.

### 3.3. Effect of Myricetin on Biofilm Formation Using the CCK-8 Assay

**Figure 2:**
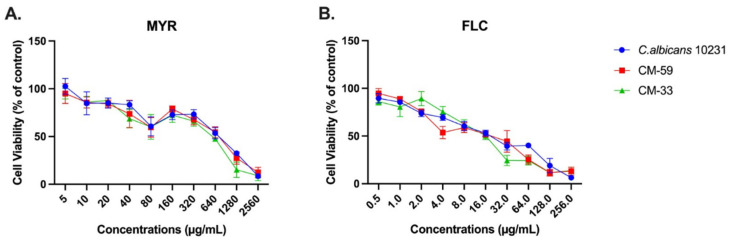
Percentage of cell viability of *C. albicans* biofilms by CCK8 assay at 24 h; (**A**) MYR, (**B**) FLC.

The cytotoxicity of MYR and FLC at different concentrations (5–2560 and 0.5–256 µg/mL, respectively) on CM-33, CM-59, and *C. albicans* ATCC 10231 was investigated for 24 h incubation, and the results obtained are shown in Figure 2. In this study, the CCK-8 assay was used to evaluate MBIC values, indicating cell viability. According to the results, myricetin exhibited biofilm inhibitory ability against CM-33, CM-59, and *C. albicans 10231.* Myricetin dose-dependently reduced the biofilm-forming ability when administered at doses ranging from 10 to 2560 µg/mL. Myricetin showed 50% inhibition against CM-33, CM-59, and *C. albicans* 10231 biofilms at 749.17, 748.52, and 599.7 µg/mL, respectively, compared to the control. Fluconazole was administered at doses between 0.5 and 256 µg mL, and the MBIC values against CM-33, CM-59, and *C. albicans* 10231 biofilms were 19.54, 20.26, and 16.2 µg/mL, respectively. To ensure dose consistency between the groups and that inhibitory concentrations were close to each other, 640 µg/mL for myricetin and 16 µg/mL for fluconazole were determined as the treatment dose for biofilm-related experiments.

### 3.4. Effect of Myricetin on Biofilm-Related Gene Expression in C. albicans

**Figure 3:**
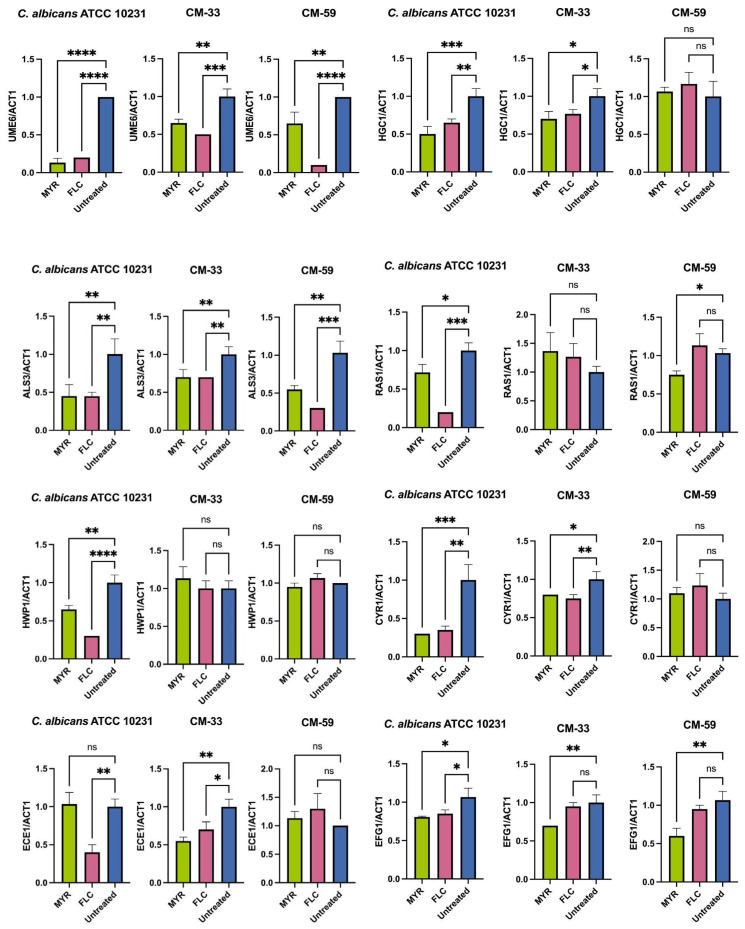
Effect of MYR and FLC on the expression of biofilm-related genes in strain CM-33, CM-59, and *C. albicans* ATCC 10231. *ACT1* ribosomal RNA was used for normalization of gene expression levels. Data are presented as mean ± SD. Statistical significance is indicated as follows: * *p* < 0.05; ** *p* < 0.01; *** *p* < 0.001; **** *p* < 0.0001; ns, no significance.

To understand the molecular mechanisms underlying biofilm formation and inhibition of hyphae growth, transcriptional changes in hyphae and biofilm-associated genes within the RAS1/cAMP/EFG1 pathway were evaluated by qRT-PCR (Figure 3). This pathway is critical in yeast hyphal growth, migration, and adhesion. The analysis was performed on reference (*C. albicans* ATCC 10231) and clinical strains (CM-33 and CM-59) using 640 µg/mL MYR and 16 µg/mL FLC.

Myricetin significantly (*p* < 0.05) reduced the expression levels of *RAS1*, *CYR1*, and *EFG1* genes in clinical and reference strains compared to the untreated control at a 640 µg/mL concentration. Furthermore, MYR also substantially down-regulated the transcription factors *UME6* [*C. albicans* ATCC 10231, CM-33 and CM-59] and *HGC1* [*C. albicans* ATCC 10231, CM-33] are important regulators of hyphal growth and filamentation (*p* < 0.05) compared to the untreated control. Furthermore, treatment with MYR also led to a significant decrease in the expression of adhesion-related genes (*ALS3* and *HWP1*) in the *C. albicans* ATCC 10231 strain.

### 3.5. Effect of Myricetin on C. albicans Biofilms by FESEM Analysis

**Figure 4:**
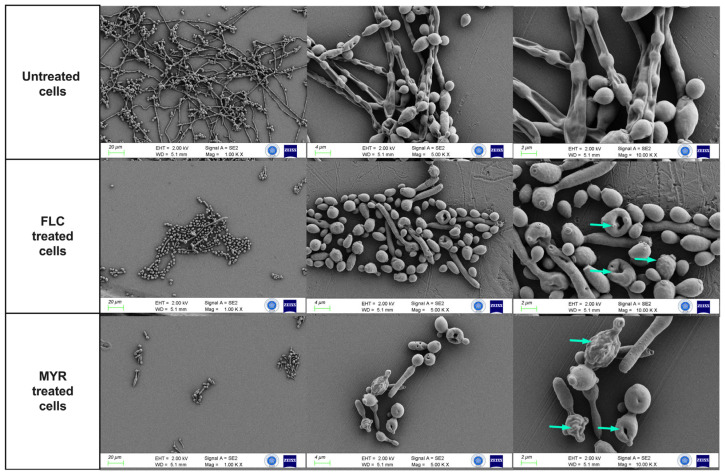
FESEM images of *C. albicans* ATCC 10231 biofilms after control and 24 h exposure to MYR and FLC. Biofilm culture treated with 640 μg/mL MYR and 16 μg/mL FLC. Magnification scales of 1000×, 5000×, and 10,000× were used for imaging.

FESEM analysis revealed that myricetin inhibits hyphal growth and development in *C. albicans*. Figure 4 shows changes in the structure of *C. albicans* (ATCC 10231) biofilm induced by 640 µg/mL MYR and 16 µg/mL FLC. Untreated controls exhibited significant hyphae formation and fusion of yeast cells with hyphae. FLC treatment decreased hyphae formation and filamentation. Myricetin substantially inhibited hyphae formation in *C. albicans* biofilms compared to the control. Furthermore, MYR formed holes in the central region of the cells, deformed the cell morphology, and caused distortions and wrinkles on the yeast cell surface (indicated by the blue arrow).

### 3.6. In Vivo Toxicity and Efficacy of Myricetin Using the Galleria Mellonella Model

**Figure 5:**
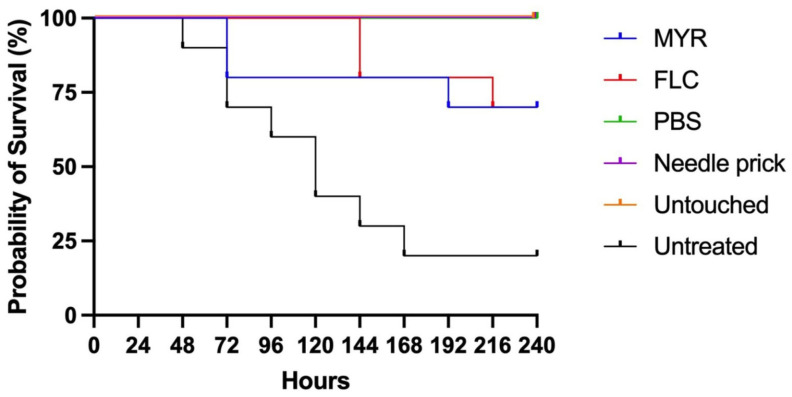
Kaplan–Meier survival curves of *G. mellonella* larvae infected with *C. albicans* ATCC 10231.

This study investigated myricetin’s in vivo toxicity and efficacy using a *G. mellonella* larvae model infected with the *C. albicans* ATCC 10231 strain. Larvae were observed daily for 10 days, and survival data were recorded and shown in Figure 5. To confirm the successful establishment of infection in the infected larvae, hemolymph samples were collected 3 h post-infection and cultured on SDA. In our study, survival rates were higher in both MYR and FLC-treated groups compared to the untreated infection group. In the myricetin group, survival rates began to decline from the third day, ultimately reaching 70% (7 out of 10 larvae) by the end of the experiment.

In contrast, the FLC group maintained a 100% survival rate for the first five days. However, from the sixth day onwards, survival rates started to decrease, reaching 70% (7 out of 10 larvae) on the final day of the experiment. In the untreated infected group, survival remained stable at 20% from the seventh day onwards. In the control groups, which were untreated healthy control (untouched), PBS-control, and needle-prick control group, survival was maintained at 100% throughout the period. These findings confirm the successful establishment of infection in the model system. While myricetin demonstrated a modest therapeutic effect, it notably improved survival rates compared to the untreated control group. The observed decrease in survival rates in the fluconazole group indicates that this antifungal agent may not be fully effective against the *C. albicans* ATCC 10231 strain. This suggests that MYR exhibits a notable therapeutic effect, though its efficacy does not reach the same level as FLC.

## 4. Discussion

Myricetin is a promising alternative as a flavonoid with natural biological properties, especially considering the potentially toxic effects of conventional antifungal agents [31,32]. Despite its potential, only a few studies have specifically investigated the antifungal activity of myricetin against *Candida* species [18,19,20,21]. The fungal cell wall and membrane are the main targets of antifungal drugs [18]. Flavonoids exhibit antifungal activity through multiple mechanisms, including cell membrane disruption, cell wall synthesis inhibition, efflux pumps, interference with RNA, DNA, and protein synthesis, and synergistic interactions with antifungal agents [33]. Lee et al. demonstrated that myricetin exhibits moderate antifungal activity against *Candida albicans* by damaging cell wall integrity and increasing membrane permeability. In this study, myricetin also altered membrane lipid components such as ergosterol, phospholipids, or sphingolipids [18].

In our study, myricetin showed significant activity against clinical and reference strains of *C. glabrata* with a low MIC_50_ value (40 μg/mL). MIC_50_ values for *C. kefyr, C. krusei, C. lusitaniae,* and *C. spherica* were 320 μg/mL, while MIC_50_ values for both clinical and reference strains of *C. albicans* and *C. tropicalis* showed a wide range from 40 to 640 μg/mL. In a study conducted by Salazar et al., MYR exhibited antifungal activity with MIC values of 3.9 μg/mL for *C. glabrata*, 16–64 μg/mL for *C. albicans*, 54 μg/mL for *C. tropicalis,* and 64 μg/mL for *C. krusei*. The antifungal activity of myricetin against clinical isolates of *C. glabrata* was found to be equal to or higher than that of FLC. Furthermore, it was observed that myricetin was 4 to 16 times more effective against *C. glabrata* than against *C. albicans* [19]. In another study investigating the antifungal activity of myricetin against *Candida* species, MIC_50_ values for *C. albicans* SC5314, *C. glabrata* ATCC 2001, *C. krusei* ATCC 6258, and *C. parapsilosis* ATCC 22019 were 20 μg/mL, 1.3 μg/mL, 5 μg/mL, and 5 μg/mL, respectively [18]. In a study investigating the synergistic effect of MYR with antifungals and its antibiofilm properties, MYR alone did not exhibit antifungal activity against *C. albicans* at concentrations between 5 and 640 µg/mL [21]. Our study found low MIC_50_ values for *C. glabrata* and 2 to 16 times higher MIC_50_ values for *C. albicans,* in line with the previous research. The enhanced antifungal activity of myricetin against *C. glabrata* has been attributed to its antioxidant properties, particularly the presence of a third hydroxyl group in its chemical structure. This structural feature plays an essential role in the mechanism of action against this specific *Candida* species [19].

Myricetin showed a synergistic effect when combined with antifungals, and its combined use significantly reduced antifungal MICs. This synergy was attributed to the ability of myricetin to increase the efficacy of antifungals by disrupting biofilm formation. In a study by Mo et al., MYR showed no antifungal activity below 640 µg/mL, whereas it inhibited biofilm formation at concentrations of 80 µg/mL or higher [21]. Another study investigated the effects of topical treatments containing myricetin + t-farnesol + fluoride on dual-species biofilms formed by *Streptococcus mutans* and *C. albicans*. Briefly, myricetin monotherapy containing two mM (approximately 636 µg/mL) was ineffective, but myricetin combination therapy effectively reduced water-soluble exopolysaccharides in the extracellular matrix, similar to chlorhexidine. Their study suggested that higher concentrations of myricetin may be required for better biofilm control [20]. The precise molecular mechanism by which MYR inhibits biofilm formation remains unclear.

The yeast-to-hyphal transition in C. *albicans* is known to be crucial for biofilm development and contributes significantly to its virulence [34]. This morphogenetic process is suggested to be regulated by several signaling pathways, including the RAS1-cAMP-EFG1 pathway [35]. The RAS1-cAMP-EFG1 pathway is activated directly by the adenylyl cyclase *CYR1* or indirectly through the small GTPase *RAS1*, stimulating *CYR1* in response to certain stimuli. Activation of this pathway phosphorylates the transcription factor *EFG1* and other downstream transcription factors such as *ALS3*, *HWP1*, and *ECE1* [35,36]. *EFG1* activation triggers a series of events, simultaneously stimulating several transcription factors involved in biofilm formation. Moreover, *EFG1* promotes the expression of numerous hyphal-specific genes by activating the transcription factor *UME6* [11,36]. Hyphal-specific G1 cyclin (*HGC1*) plays an important role in hyphal morphogenesis, and *UME6* regulates its expression. Furthermore, *HGC1* has been shown to influence the activity of both *EFG1* and *UME6*, thus forming a complex regulatory network governing hyphal growth and, consequently, biofilm formation [36,37]. Understanding these pathways is essential for elucidating possible mechanisms explaining how myricetin may interfere with *C. albicans* biofilm development.

In a recent study, transcription patterns of three genes (*TPK2*, *EFG1*, and *HST7*) were analyzed by qRT-PCR assay to evaluate the effect of MYR on the yeast-to-hyphae transition of *C. albicans*. Expression levels of *TPK2*, *EFG1*, and *HST7* were significantly down-regulated in *C. albicans* treated with MYR (80 μg/mL). Furthermore, MYR was reported to inhibit biofilm formation by interfering with the transition from *C. albicans* yeast to hyphae [21]. Similar to this study, our study showed that MYR (640 μg/mL) significantly down-regulated the expression of hyphal cAMP-dependent protein kinase regulators (*RAS1*, *CYR1*, *EFG1*) genes in *C. albicans* 10231 and clinical strain (CM-33) compared to untreated (*p* < 0.05). In addition, the expression of genes related to adhesion (*ALS3*, *HWP1*, and *ECE1*) and hyphal development (*UME6* and *HGC1*) was also significantly down-regulated in both the standard and clinical strain (CM-33).

As part of our study, FESEM was also used to examine the effects of myricetin on *C. albicans* morphology. Extensive hyphal formation and yeast-hyphal fusion were observed in untreated controls. Fluconazole (16 µg/mL) reduced hyphae formation, while myricetin (640 µg/mL) showed a potent inhibitory effect on *C. albicans* hyphae in biofilms. Myricetin also caused significant structural damage to yeast cells, including central holes, surface distortions, and wrinkles. Consistent with our study, the effect of MYR on *C. albicans* biofilm was investigated by scanning electron microscopy (SEM), and MYR (80 µg/mL) can suppress dense biofilm formation. Moreover, when combined with other antifungals, MYR caused a significant decrease in biofilm thickness and total biofilm mass [21].

Our study evaluated the in vivo activity of MYR against the *C. albicans* ATCC 10231 strain using a *G. mellonella* larval model. As shown in previous studies, the *G. mellonella* model is considered an alternative in vivo model to assess the toxicity and efficacy of antimicrobial agents before experimental animal models [38]. Myricetin did not show any toxic effect up to a concentration of 50 mg/kg in a study evaluating the antimicrobial impact of MYR using *G. mellonella* larvae in *Staphylococcus aureus* infection [39]. However, to the best of our knowledge, there is no study evaluating the antifungal activity of MYR in *Candida* infection in *G. mellonella* larvae. In our study, the survival rate of 70% in the myricetin-treated group showed a significant improvement compared to the untreated infected group (20%). These findings support the therapeutic potential of myricetin against *C. albicans* infections in vivo. The protective effect of MYR declined from the third day of treatment, compared to FLC treatment, which provided 100% survival during the first five days. This suggests that FLC shows a faster and stronger antifungal effect in the early stages of infection. However, at the end of the experiment, both treatment groups achieved a 70% survival rate, suggesting FLC was not fully effective against *C. albicans* ATCC 10231.

Our study has several limitations. One limitation of our study is that despite myricetin’s promising antibiofilm properties, its antifungal potential is limited by the high MIC values observed. Many strains required MIC_50_ concentrations as high as 640 µg/mL, which may restrict their clinical application unless pharmacological optimization or improved delivery strategies are developed. Another limitation of our study is the relatively small sample size used in the *Galleria mellonella* infection model. Although this invertebrate system provides valuable preliminary in vivo data, the use of only 10 larvae per experimental group limits the statistical power of our findings. Future studies should use larger groups to strengthen the reliability of survival outcomes.

## 5. Conclusions

This study demonstrates myricetin’s antifungal and antibiofilm activity against both fluconazole-resistant and susceptible *Candida* strains. Significant suppression of critical genes involved in hyphae development (*RAS1*, *CYR1*, *EFG1*, *UME6*, and *HGC1*) and adhesion genes (*ALS3* and *HWP1*) by myricetin at a concentration of 640 µg/mL indicates the molecular basis of its antibiofilm mechanism of action. FESEM analyses confirmed that myricetin inhibited hyphae growth and development in *C. albicans*. This finding agrees with gene expression analyses and supports the ability of myricetin to inhibit yeast-hyphae transition. The improvement observed in the survival rate of larvae in the in vivo activity evaluation performed in the *G. mellonella* model supports the therapeutic potential of myricetin.

In conclusion, the antifungal and antibiofilm properties of myricetin may be mediated by its inhibitory effect on the RAS1/cAMP/EFG1 pathway. These findings emphasize myricetin’s therapeutic potential in treating *Candida* infections and the importance of natural active ingredients in developing new strategies. Future studies may include the evaluation of different doses and protocols of myricetin, as well as the assessment of efficacy on different *Candida* species and murine models.

## Figures and Tables

**Table 1:** List of primers used in this study.

Genes	Primer	Sequence (5′ ⟶ 3′)	Reference
*ALS3*	Forward	TCGTCCTCATTACACCAACCA	Sumlu et al., 2024 [13].
	Reverse	TGAAGTTGCAGATGGGGCTT	
*HWP1*	Forward	GCTCCTGCTCCTGAAATGAC	Holcombe et al., 2010 [26]
	Reverse	CTGGAGCAATTGGTGAGGTT	
*ECE1*	Forward	TTGCTAATGCCGTCGTCAGA	Willems et al., 2018 [27]
	Reverse	GAACGACCATCTCTCTTGGCAT	
*RAS1*	Forward	TGGATGTTGTGTTATTGTTTGAGC	Sumlu et al., 2024 [13]
	Reverse	GTCTTGAATTGTTCATCTTCTCCCA	
*CYR1*	Forward	CCAACAAACGACCAAAAGGT	Hsu et al., 2013 [28]
	Reverse	TCTTGAACTGCCAGACGATG	
*EFG1*	Forward	GCCTCGAGCACTTCCACTGT	Uppuluri et al., 2009 [29]
	Reverse	TTTTTTCATCTTCCCACATGGTAGT	
*UME6*	Forward	ACCACCACTACCACCACCAC	O’Connor et al., 2010 [30]
	Reverse	TATCCCCATTTCCAAGTCCA	
*HGC1*	Forward	GCTTCCTGCACCTCATCAAT	Hsu et al., 2013 [28]
	Reverse	AGCACGAGAACCAGCGATAC	
*ACT1*	Forward	TTTCATCTTCTGTATCAGAGGAACTTATTT	Sumlu et al., 2024 [13]
	Reverse	ATGGGATGAATCATCAAACAAGAG	

**Table 2:** MIC_50_ range of fluconazole (FLC) and myricetin (MYR) to *Candida* strains.

Species (Number)	MIC_50_ Range of FLC (µg/mL)	MIC_50_ Range of MYR (µg/mL)
*C. albicans* ATCC 10231	0.5	320
*C. krusei* ATCC 6258	32	80
*C. parapsilosis* ATCC 22019	16	40
*C. glabrata* ATCC 90030	16	40
*C. albicans (10)*	0.5–16	40–640
*C. glabrata (4)*	2–16	40
*C. tropicalis (4)*	2–16	40–640
*C. kefyr (4)*	1	320
*C. spherica (2)*	2	320
*C. krusei (2)*	32–128	320
*C. lusitaniae (2)*	0.5	320

## Data Availability

The data presented in this study are available on request from the corresponding author due to privacy.

## Supplementary Materials

The following supporting information can be downloaded at https://www.mdpi.com/article/10.3390/jof11050398/s1: Table S1: MIC_50_ values of clinical and reference *Candida* spp. strains.
