# Supplementary material for: Myricetin Exerts Antibiofilm Effects on Candida albicans by Targeting the RAS1/cAMP/EFG1 Pathway and Disruption of the Hyphal Network
Source: J Fungi (Basel). 2025 May 21;11(5):398. doi: 10.3390/jof11050398 (PMC12113504; doi:10.3390/jof11050398)
Supplement: Supplementary file 1 [file jof-11-00398-s001.zip › jof-3617939-supplementary.pdf]

Myricetin exerts antibiofilm effects on *Candida albicans* by targeting the RAS1/cAMP/EFG1 pathway and disruption of the hyphal network

Melda Meral Ocal[1], Merve Aydin[2,3*], Esra Sumlu[4], Emine Nedime Korucu[5], Ali Ozturk[6]

[1] Department of Biotechnology, Faculty of Science, Mersin University, 33343, Mersin, Turkey; meldameralocal@mersin.edu.tr
[2] Department of Medical Microbiology, Faculty of Medicine, Erzincan Binali Yildirim University, 24100, Erzincan, Turkey; merve.terzioglu@erzincan.edu.tr
[3] Department of Medical Microbiology, Faculty of Medicine, KTO Karatay University, 42020, Konya, Turkey
[4] Department of Medical Pharmacology, Faculty of Medicine, KTO Karatay University, 42020, Konya, Turkey; esra.sumlu@karatay.edu.tr
[5] Department of Molecular Biology and Genetics, Faculty of Science, Necmettin Erbakan University, 42090, Konya, Turkey; enkorucu@erbakan.edu.tr
[6] Department of Medical Microbiology, Faculty of Medicine, Nigde Omer Halisdemir University, 51240, Nigde, Turkey; ozturkali@ohu.edu.tr
* Correspondence: merve.terzioglu@erzincan.edu.tr; Tel.: +90-(446)-226-18-18-31159

Table S1. MIC_{50} values of clinical and reference *Candida* spp. strains.

Strain no	*Candida species*	FLC (µg/ml)	MYR (µg/ml)
CM-33	*C. albicans*	16	640
CM-41	*C. albicans*	16	640
CM-59	*C. albicans*	1	640
CM-62	*C. albicans*	16	40
CM-73	*C. albicans*	1	640
CM-78	*C. albicans*	0,5	320
CM-85	*C. albicans*	2	320
CM-89	*C. albicans*	16	40
CM-108	*C. albicans*	16	320
CM-217	*C. albicans*	16	80
CM-23	*C. glabrata*	8	40
CM-25	*C. glabrata*	16	40
CM-47	*C. glabrata*	16	40
CM-75	*C. glabrata*	2	40
CM-1	*C. kefyr*	1	320
CM-8	*C. kefyr*	1	320
CM-13	*C. kefyr*	1	320
CM-101	*C. kefyr*	1	320
CM-11	*C. spherica*	2	320
CM-221	*C. spherica*	2	320
CM-57	*C. krusei*	128	320
CM-176	*C. krusei*	32	320
CM-83	*C. lusitaniae*	0,5	320
CM-100	*C. lusitaniae*	0,5	320
CM-29	*C. tropicalis*	16	40
CM-48	*C. tropicalis*	16	40
CM-55	*C. tropicalis*	2	320

CM-58	*C. tropikalmiş*	2	640
Reference strain	*C. albicans ATCC 10231*	0,5	320
Reference strain	*C. krusei ATCC 6258*	32	80
Reference strain	*C. parapsilosis ATCC 22019*	16	40
Reference strain	*C. glabrata ATCC 90030*	16	40

The antifungal activity test was repeated three times for each strain. The minimum inhibitory concentration (minimum inhibitory concentration, MIC50) values for MYR and FLC were determined visually by two blinded observers and defined as the lowest concentration of the drugs that resulted in 50% growth inhibition as relative to the growth of the control well. SD was not calculated as the results were obtained through visual reading.
